# C-1101 multi-protein platelet and plasma-derived therapeutic to treat chronic lumbosacral radiculopathy

**DOI:** 10.3389/fbioe.2026.1918730

**Published:** 2026-09-08

**Authors:** Peter Linde, Lyndah Chow, Renata Impastato, Teresa Wright, Vishwesh Patil, Jennifer Schmitke, Steven Dow, Lynn M. Pezzanite

**Affiliations:** 1 Orthopaedic Research Center, Department of Clinical Sciences, College of Veterinary Medicine and Biomedical Sciences, Translational Medicine Institute, Colorado State University, Fort Collins, CO, United States; 2 Immunotherapy Research Laboratory, Department of Clinical Sciences,Translational Medicine Institute, College of Veterinary Medicine and Biomedical Sciences, Colorado State University, Fort Collins, CO, United States; 3 Consano Bio, Burlington, MA, United States

**Keywords:** chronic lumbosacral radiculopathy, degenerative disc disease, orthobiologic, pain, spine, chronic sciatica

## Abstract

**Introduction:**

Intervertebral disc disease (IVDD) leads to chronic lumbosacral radiculopathy (LSR) with nerve root compression resulting in numbness and low back pain. The aim was to evaluate therapeutic and immunomodulatory potential of multi-protein platelet and plasma-derived biologic (C-1101) to alter inflammatory mediators and gene expression in IVDD.

**Methods:**

In aim 1, intervertebral discs and dorsal root ganglia (DRG) were harvested from female ovine lumbar spines (n = 6). Annulus fibrosus (AF), nucleus pulposus (NP) and DRG were dissociated to form single-cell suspensions. Cells were plated (100,000 cells/well, 24-well plates) and subjected to 4 treatments, 24 h (non-stimulated, IL-1β+TNF-α, IL-1β+TNF-α+C-1101, IL-1β+TNF-α+vehicle). Cells were washed, cultured 24 h, and media evaluated for cytokine secretion (n = 14) by ovine multiplex immunoassay. mRNA was collected from cells and sequencing performed via Illumina-based platform. In aim 2, peripheral neuropathy was induced (paclitaxel) in male C57Bl6/J mice who were treated intravenously with C-1101 or vehicle. Mice were assessed via acetone cooling for allodynia and plasma evaluated for cytokines.

**Results:**

C-1101 elicited cytokine release from ovine AF and NP cells but not DRG. Gene expression analyses revealed upregulation of cell replication and translation pathways but downregulation of inflammatory (namely, interferons), extracellular matrix and cell signaling pathways in AF/NP, with upregulation of translation and ribosome activity in DRG. C-1101 reduced allodynia and transiently induced elevated plasma IL-1β in murine peripheral neuropathy.

**Discussion:**

Functional reduction in pain sensation with C-1101 was observed in murine peripheral neuropathy, indicating potential therapeutic efficacy. C-1101 induced a mixed inflammatory response in cultured cells, warranting further investigation of mechanism.

## Introduction

The prevalence of musculoskeletal conditions, particularly low back pain (LBP), is growing globally, affecting ∼80% of adults at some point in their lives (Freburger et al., 2009; De David et al., 2020). The etiology of LBP is multifactorial, with 40% of cases linked to degenerative disc disease (DDD) at an economic cost in the US alone exceeding $100 billion annually (Freburger et al., 2009; Freemont et al., 2002; Binch et al., 2015; Freemont et al., 1997; Zhang et al., 2009; Dagenais et al., 2008; Hartvigsen et al., 2018). DDD is characterized by a progressive loss of structural integrity, inflammation, and impaired function in disc components including nucleus pulposus and annulus fibrosis, while triggering inflammation in associated dorsal root ganglia (Antoniou et al., 1996; Ciapetti et al., 2012; Diwan and Melrose, 2022; Setton and Chen, 2006; Machino et al., 2021; Kos et al., 2019). Structural damage in DDD (*i.e.,* disc protrusion, herniation, or degenerative vertebral deformations) may lead to nerve root compression and associated inflammatory mediator release resulting in lumbosacral radiculopathy (LSR) also known as sciatica, ischias or nerve root pain (Backonja et al., 2017; Alexander et al., 2025). Generalized radicular pain is one of the most common forms of neuropathic pain with an estimated lifetime incidence of 10% to 40% and prevalence up to 43% (Davis et al., 2025; Konstantinou and Dunn, 2008).

Current clinical treatments for LSR aim to primarily manage symptoms, relying on physical therapy and pharmacological approaches, including non-steroidal anti-inflammatory drugs (NSAIDs), corticosteroids, and opioids. Among the numerous non-surgical interventions, high-dose epidural steroid injections (ESI) are commonly performed to manage chronic low back and lower extremity pain (Rosas and Lee, 2012). When conservative treatments fail, surgical options like spinal fusion or total disc arthroplasty may be used (Costăchescu et al., 2022; Romaniyanto et al., 2022). While these treatments can provide transient symptomatic relief, they may not preserve disc function and can accelerate adjacent-level degeneration, failing to address the underlying mechanisms of degeneration and progression (Alizadeh and Sharifzadeh, 2022).

Regenerative therapies represent an expanding area of research, aiming to modify the inflammatory environment and promote tissue repair in DDD. However, unique challenges of disc degeneration, including poor vascularization and complex inflammatory cascades, have resulted in complicated clinical translation (Farag et al., 2024). Platelet-derived biologics have emerged as promising regenerative therapies in orthopedics and spine medicine, leveraging concentrated growth factors (PDGF, TGF-β, VEGF, IGF, EGF) and anti-inflammatory cytokines naturally stored in platelet α-granules to promote tissue repair and modulate inflammation. Platelet lysate (PL) offers distinct advantages over traditional platelet-rich plasma as it provides immediate release of growth factors without requiring activation, exhibits superior immunomodulatory properties suitable for use around neural structures and has demonstrated significant pain reduction and functional improvement (Meftahpour et al., 2021; Rawson, 2020). The novel C-1101 multi-protein, platelet and plasma-derived product is a promising therapeutic option to potentially harness the innate healing capabilities of platelets but requires further investigation to treat LSR.

This exploratory study investigated impact of C-1101 on key cellular components of neuropathic pain pathophysiology. The aims were to define effect on cytokine secretion and differential gene expression from three relevant spine tissue derived cell lines (nucleus pulposus, annulus fibrosis, dorsal root ganglia) using a relevant large animal (ovine) model, and reduction of allodynia in a rodent model of peripheral neuropathy. The hypothesis was that C-1101 would have a multi-modal mechanism of action involving immunomodulation and growth factor activity, resulting in downregulation of inflammatory pathways such as interferons with differential gene expression patterns favoring tissue repair and pain modulation that could contribute to a reparative cascade relevant to treating LSR.

## Methods

### Study overview

**FIGURE 1:**
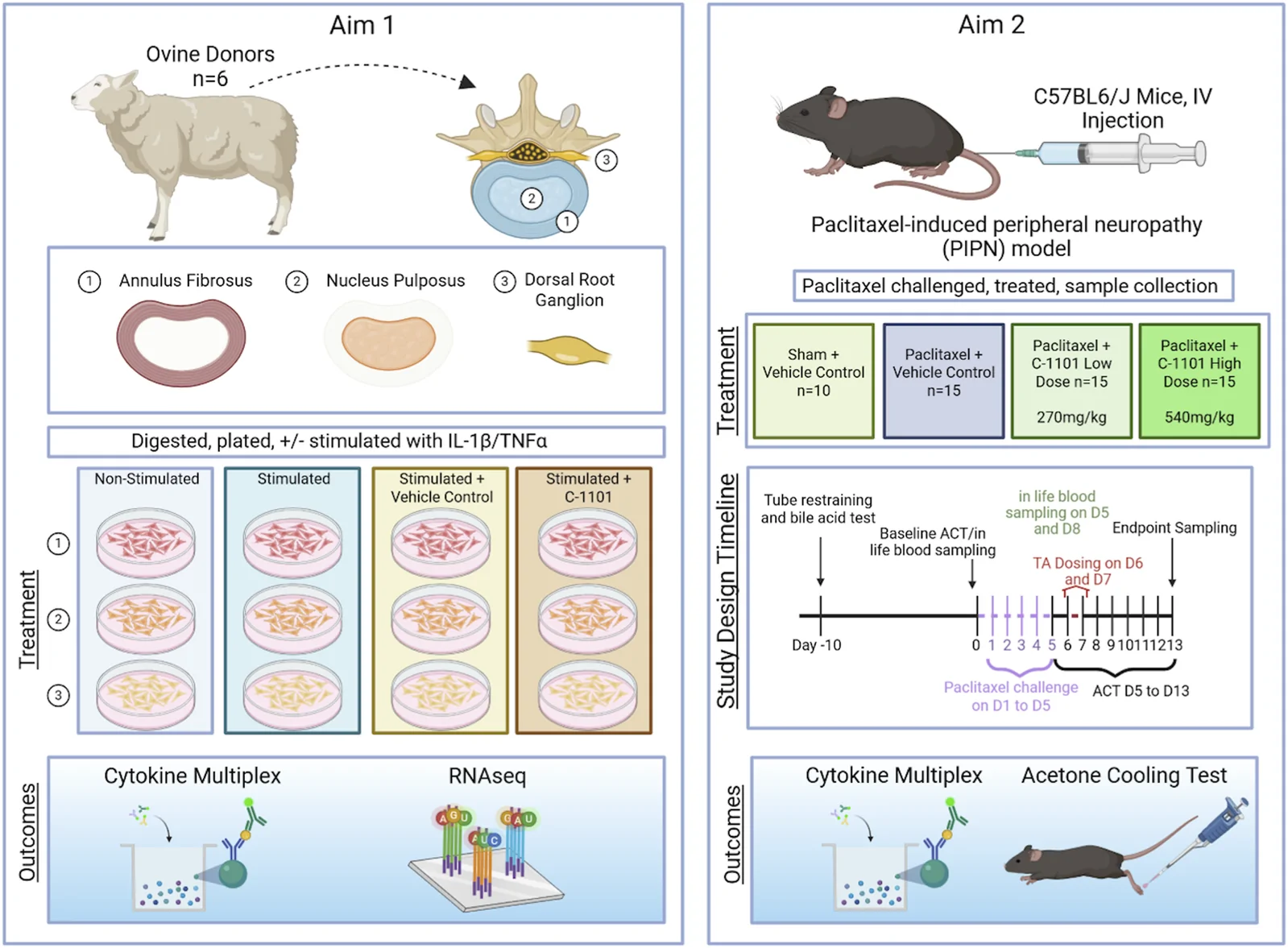
Study overview. In aim 1, adult sheep (n = 6 total) were donors of lumbar spines for annulus fibrosus, nucleus pulposus, and dorsal root ganglia cell culture, which were treated with C-1101 platelet lysate product in inflammatory culture conditions. Cellular RNA was submitted for transcriptomic analyses to determine gene expression following treatment, and conditioned media were generated as described below. Quantitative analysis of cytokines was measured in conditioned media from treated cells. In aim 2, chemotherapy (paclitaxel) peripheral neuropathy was induced in male C57Bl6/J mice who were treated intravenously with C-1101 or vehicle control. Mice were assessed via acetone cooling test for allodynia, cytokine analyses of plasma and Iba1 immunohistochemistry staining of sciatic nerves.

The Institutional Animal Care and Use Committee at Colorado State University (No. CSU IACUC #5264) and Charles River Discovery Services, Finland (IACUC C2570123 and C3140124) approved ovine tissue collection and murine *in vivo* studies, respectively. All methods were conducted according to the national guidelines under which the institution operates and National Institute of Health (NIH) Guidelines for the Care and Use of Laboratory Animals (eighth edition). In **aim 1**, adult sheep (n = 6 total) were donors of lumbar spines for annulus fibrosus, nucleus pulposus, and dorsal root ganglia cell culture, which were treated with C-1101 in inflammatory culture conditions. Cellular RNA was submitted for transcriptomic analyses to determine gene expression following treatment, and conditioned media were generated as described below. Quantitative analysis of cytokines was measured in conditioned media from treated cells. In **aim 2**, chemotherapy (paclitaxel) peripheral neuropathy was induced in male C57BL6/J mice who were treated intravenously with C-1101 or vehicle control. Mice were assessed via acetone cooling test for allodynia and cytokine analyses of plasma were performed. An overview of the experimental workflow is shown in Figure 1.

### C-1101 manufacturing

C-1101 is manufactured from pooled, transfusable human platelets collected by a Food and Drug Administration (FDA)-registered, Association for the Advancement of Blood and Biotherapies (AABB)-accredited US blood center. (For the Submission of Chemistry, 2026) Blood is collected from healthy donors following established procedures for platelet unit collection, storage, and testing in accordance with current FDA guidelines and regulations. Eligibility of donors is performed according to 21 CFR 630.10 and 640.21 following a donor questionnaire. C-1101 is composed of plasma proteins (including but not limited to HSA, and immunoglobulins), and platelet proteins (such as platelet-derived growth factor [PDGF]), as well as other growth factors and cytokines including transforming growth factor beta 1 [TGF-β1]). C-1101 has been designed to retain the benefits observed from autologous platelet-based treatments, and to improve upon them by providing a consistent, defined, and controlled product.

### Ovine intervertebral disc cell isolation

Annulus fibrosus (AF) and nucleus pulposus (NP) cells were obtained from intervertebral discs (IVDs) from the spine. Briefly, IVDs were excised from between lumbar vertebral bodies, AF and NP were separated, minced and digested for 2 h in complete media (Dulbecco’s Modified Eagle Medium, 10% fetal bovine serum (FBS), penicillin (100units/mL), streptomycin (100 ug/mL), 1M HEPES ((4-(2-hydroxyethyl)-1-piperazineethanesulfonic acid))) with pronase (0.4%). After 2 h, pronase-supplemented media was removed, and tissues were further digested overnight in complete media with type II collagenase (0.2%). Digested tissue was filtered through 70 uM and 40 uM cell strainers (Greiner Bio-one), where cells were counted, plated and expanded to second passage, then cryopreserved in freeze media (90% FBS, 10% DMSO). For treatment, AF and NP cells were thawed in 37° C water bath, plated and recovered overnight in complete media. Then, cells were plated at 100,000 per well in a 24 well cell culture plate.

### Ovine dorsal root ganglia cell dissociation

Dorsal root ganglia cells were obtained from the lumbar spine; soft tissues were dissected to expose the lumbar spine and dorsal portion each vertebrae was removed to expose the spinal cord and DRGs. DRGs were separated from the spinal cord, minced, and dissociated overnight in complete media supplemented with dispase (5 units/ml) and type II collagenase (10,000 units/ml) in a protocol adapted from prior publications (Smith et al., 2023; Perner and Sokol, 2021) based on visual examination and serial cell counting and viability testing during the dissociation step. Cells were counted and suspended in complete media then plated at 100,000 per well in a 24 well cell culture plate to assess treatment effect.

### Ovine cell population characterization

Surgical extraction was performed meticulously and only the structures/areas indicated on the protocol were included in the cell cultures. Although the cell populations were not characterized by surface markers, using published single cell data on these different cell types, a gene list was constructed to further identify the AF, NP and DRGs (Usoskin et al., 2015; Avraham et al., 2022; Risbud et al., 2015; Malin et al., 2007). The following are the gene lists used to identify the subtypes. A heatmap of expression levels is provided as a supplemental document for review (Supplementary Document S1).

Sensory_Neurons: (“TUBB3”, “RBFOX3”, “NEFH”, “SCN9A”, “TRPV1”)

Satellite_Glia: (“FABP7”, “KCNJ10”, “S100B”, “GFAP”, “PLP1”)

Schwann_Cells: (“MPZ”, “MBP”, “NCAM1”, “SOX10”, “EGR2”)

Endothelial_Pericytes: (“PECAM1”, “CD34”, “PDGFRB”)

NP_Cells: (“ACAN”, “COL2A1”, “KRT19”, “KRT8”, “PAX1”, “FOXF1”, “TBXT”, “CD24”)

AF_Cells: (“COL1A1”, “COL1A2”, “MGP”, “DCN”, “THBS2”)

Macrophages: (“CD68”, “AIF1”, “CSF1R”, “CD14”, “ITGAM”)

Leukocytes: (“PTPRC”)

Fibroblasts: (“DCN”, “PDGFRA”, “FAP”, “THY1”, “ACTA2”)

### Ovine cells *in vitro* treatment with C-1101

To assess impact of C-1101 treatment, AF, NP, and DRG cells were plated at 100,000 cells per well. Cells were then stimulated with interleukin-1β (IL-1β, 20 ng/mL, PeproTech) and tumor necrosis factor (TNF-α 20 ng/mL, Kingfisher Biotech) based on previous dose titration studies performed in large animal preclinical musculoskeletal tissue models, retaining cell viability >97% (Linde et al., 2025). Cells were treated with C-1101 in complete culture media with heparin to eliminate clotting (20 units/mL, Sagent Pharmaceuticals). The stimulated and treated cells were cultured for 24 h, then washed with PBS, and either harvested for RNA collection or cultured for an additional 24 h in complete media to condition culture media for cytokine analysis. For RNA collection, cells were collected in RLT lysis buffer and frozen at −20° C until RNA isolation was performed. For cytokine analysis, cell conditioned supernatant was collected and frozen at −20° C until the multiplex bead assay was performed. Experimental groups include control (complete media with heparin), stimulated (complete media with heparin, IL-1β and TNF-α)), vehicle (complete media with heparin, IL-1β, TNF-α, and a volume of vehicle [10 mM sodium phosphate, 0.2 mM EDTA, 10% sucrose, pH 7.5] equivalent to C-1101 volume (117 uL per 350 uL)), and C-1101 treatment (complete media with heparin, IL-1β, TNF-α, and C-1101 (50 mg/mL)). C-1101 dose (50 mg/mL) was based on dose titration experiments (range 1–50 mg/mL) demonstrating that 50 mg/mL elicited a robust cytokine response (*e.g.,* VEGFa, IL-10) from AF cells above vehicle control without reduction in cell viability <95% (Supplementary Document S1).

### Ovine cell culture supernatant cytokine quantification

Fluorescent bead-based multiplex assay (Milliplex MAP Ovine Cytokine/Chemokine Magnetic Beads Multiplex Assay, Millipore Sigma) was used to quantify the concentrations of 14 analytes [IFNγ, IL-1α, IL-1β, IL-4, IL-6, IL-8 (CXCL8), IL-10, IL-17A, IL-36RA (IL-1F5), IP-10 (CXCL10), MIP-1α (CCL3), MIP-1β (CCL4), TNFα, VEGF-A] in cell culture supernatants following manufacturer’s instructions.

### Ovine RNA isolation and mRNA sequencing

Total RNA was extracted from the nucleus pulposus, annulus fibrosus, and dorsal root ganglion cells using the RNeasy® Mini Kit (Qiagen) according to the manufacturer’s instructions. An on-column DNase digestion was performed using the RNase-Free DNase Set (Qiagen) to remove genomic DNA. The quantity and integrity of the isolated RNA were assessed using the Agilent 4150 TapeStation (Agilent Technologies, Inc.) with the High Sensitivity RNA ScreenTape Assay and buffer. While AF and NP RNA were adequate (n = 6 each), low RNA content in dorsal root ganglia samples required two DRG samples combined into one for analysis (n = 3). Samples with an RNA Integrity Number (RIN) of ≥6.5 were deemed acceptable for library preparation and subsequent sequencing. Samples were sequenced at Novogene, Inc. Novogene protocols included poly-dt magnetic bead filtering for mRNA, followed by library prep using ABclonal mRNA-seq Lib Prep Kit, then 150bp paired end sequencing on the Illumina NovaSeq X Plus.

### Systemic evaluation of efficacy in murine model of peripheral neuropathy

Chemotherapy (paclitaxel)-induced peripheral neuropathy (PIPN) (Klein and Lehmann, 2021) was induced in male C57BL6/J mice (sham group 10 mice/group, paclitaxel + vehicle or C-1101 15 mice/group) to assess effect of systemic C-1101 administration on allodynia, and systemic cytokine levels (Charles River Discovery Services, Finland). Animals were randomly assigned to the treatment Groups using a stratified randomization scheme designed to achieve balanced groups based on baseline body weight and pre-treatment ACT score. Test article administration was performed by investigators blinded to the treatment. Group size was selected upon the experience and recommendation of the laboratory for this model (Charles River Discovery Services, Finland) and confirmed with a pilot study of similar size (data not shown). Male mice were evaluated initially to reduce overall numbers of animals while achieving sufficient sample size to evaluate behavioral endpoints. Paclitaxel or vehicle (12.5% Kolliphor EL, 12.5% ethanol, 75% saline) was administered intraperitoneally (15 mg/kg/day, days 1–5). C-1101 was administered intravenously (IV 270 or 540 mg/kg/day or vehicle, days 6,7). Acetone cooling test (ACT) (Knowlton et al., 2011) was performed at baseline, and daily (day 5–13) to assess allodynia. Murine plasma was collected at 4 timepoints (baseline, day 5, day 8, and endpoint) and analysed by cytokine multiplex IL-1β, IL-6, IL-10, TNF-α, IFN-γ (MILLIPLEX MAP Mouse Cytokine/Chemokine Magnetic Bead Panel, Custom Premix MCYTOMAG-70K).

Microglial activation was measured by Iba-1 (Ionized calcium-binding adapter molecule) IHC staining and evaluated from the D13 endpoint sciatic nerve. The immunostaining was performed with an automated IHC staining system (Ventana Discovery Ultra, Roche) on 5 µm thick paraffin sections paraffin sections. The primary antibody was rabbit anti-Iba-1 (Wako, Catalogue number 019–19741). The analysis was performed for Iba-1 IHC for whole section area and results were reported as positive staining percentage of region area (%): [%area = (stained area/total analyzed region area) * 100)].

## Data analysis

Cytokine and Iba1 immunohistochemistry data were assessed for normality via Shapiro-Wilk tests, and visual assessment of diagnostic plots. The effect of treatment on cytokine production was evaluated by one-way ANOVA with post-hoc Tukey’s adjustment for multiple comparisons (normal data) or Kruskal–Wallis test (non-normal data). Statistical analyses, graph analyses and graphical representations were performed using PRISM Software v9.1.1. Statistical significance was established as p < 0.05.

For RNA sequencing data analysis, demultiplexed fasq reads from Novogene Inc., were aligned and analyzed using the Alpine high performance computing resource at the University of Colorado Boulder Research Computing. (2023). Alpine. (Ref: University of Colorado Boulder. https://doi.org/10.25811/k3w6-pk81). Fastq files were filtered for quality and adapters removed using fastp v0.23.2, then aligned with sheep (*Ovis aries*) genome assembly ARS-UI_Ramb_v2.0 and annotated with Ensembl version 114 using STAR v 2.7.10b. Differential expression analysis computed using DEseq with biological sample pairing. Volcano plots were generated using ggplot, heatmaps with pheatmap using Rstudio (2024.04.0 + 735“Chocolate Cosmos” Release). For pathway analysis, median ratio normalized counts were used to perform GSEA with gene set permutation and weighted enrichment statistic (Hallmarks, Reactome, WikiPathways, PID, and GOBP) (Subramanian et al., 2005; Love et al., 2014; Anders et al., 2015; Martin, 2011). The datasets presented can be accessed online at NCBI GEO public genomic data repository (accession number GSE335974).

## Results

### C-1101 treatment upregulated inflammatory cytokine release from ovine IVD cells in an inflamed *in vitro* environment

**FIGURE 2:**
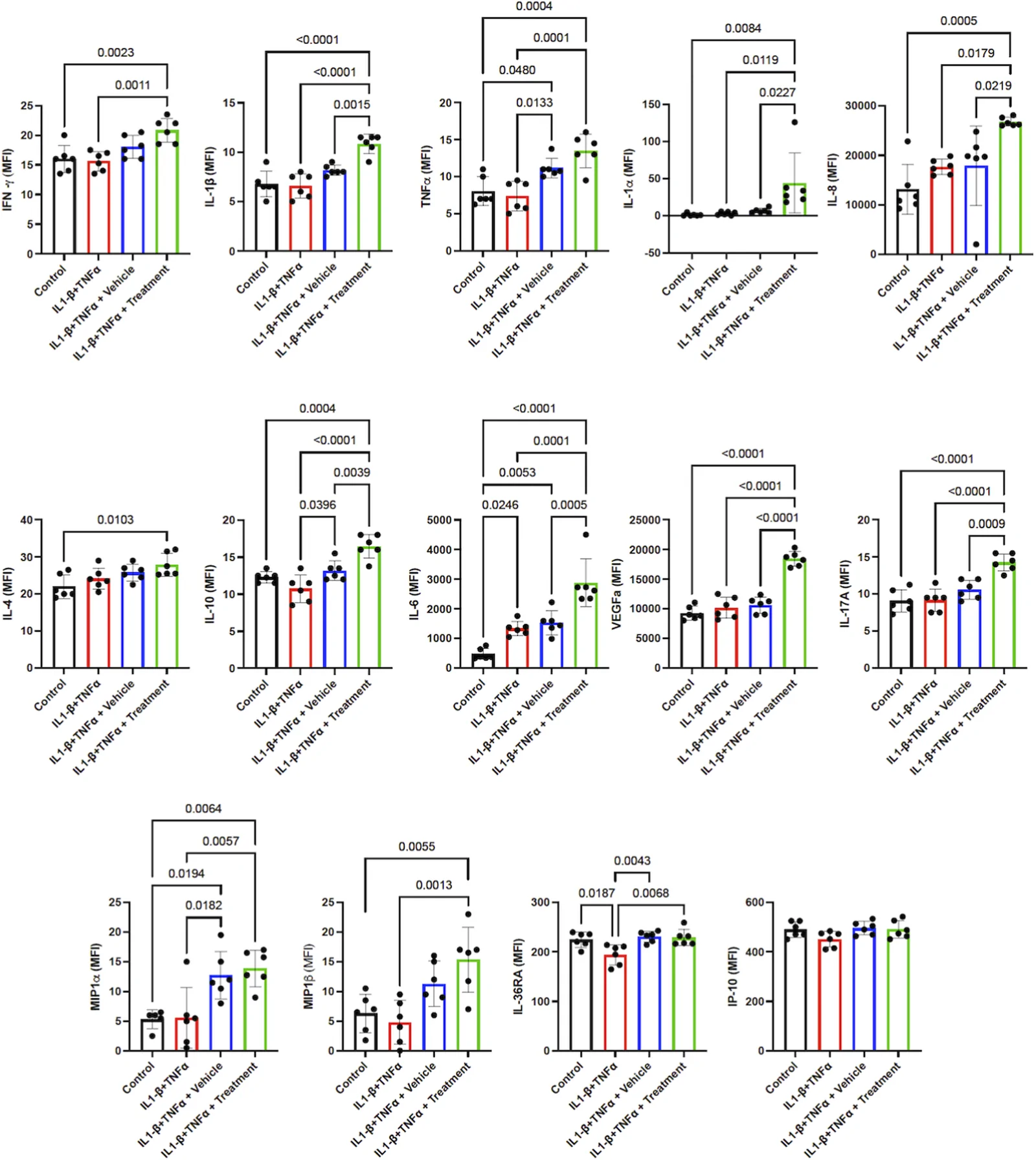
C-1101 promoted pro- and anti-inflammatory cytokine secretion in inflamed ovine annulus fibrosis (AF) cells**–**Ovine AF cells were stimulated with IL-1β and TNFα (20 ng/ml) and then treated with vehicle control or C-1101 (50 mg/ml). A non-stimulated control was also used. The stimulated and treated cells were cultured for 24 h, washed with PBS, then cultured for an additional 24 h in complete culture media. Cell-conditioned media was collected at 24 h and frozen at −20° C until it was thawed and analyzed by multiplex bead assay (14-plex).

**FIGURE 3:**
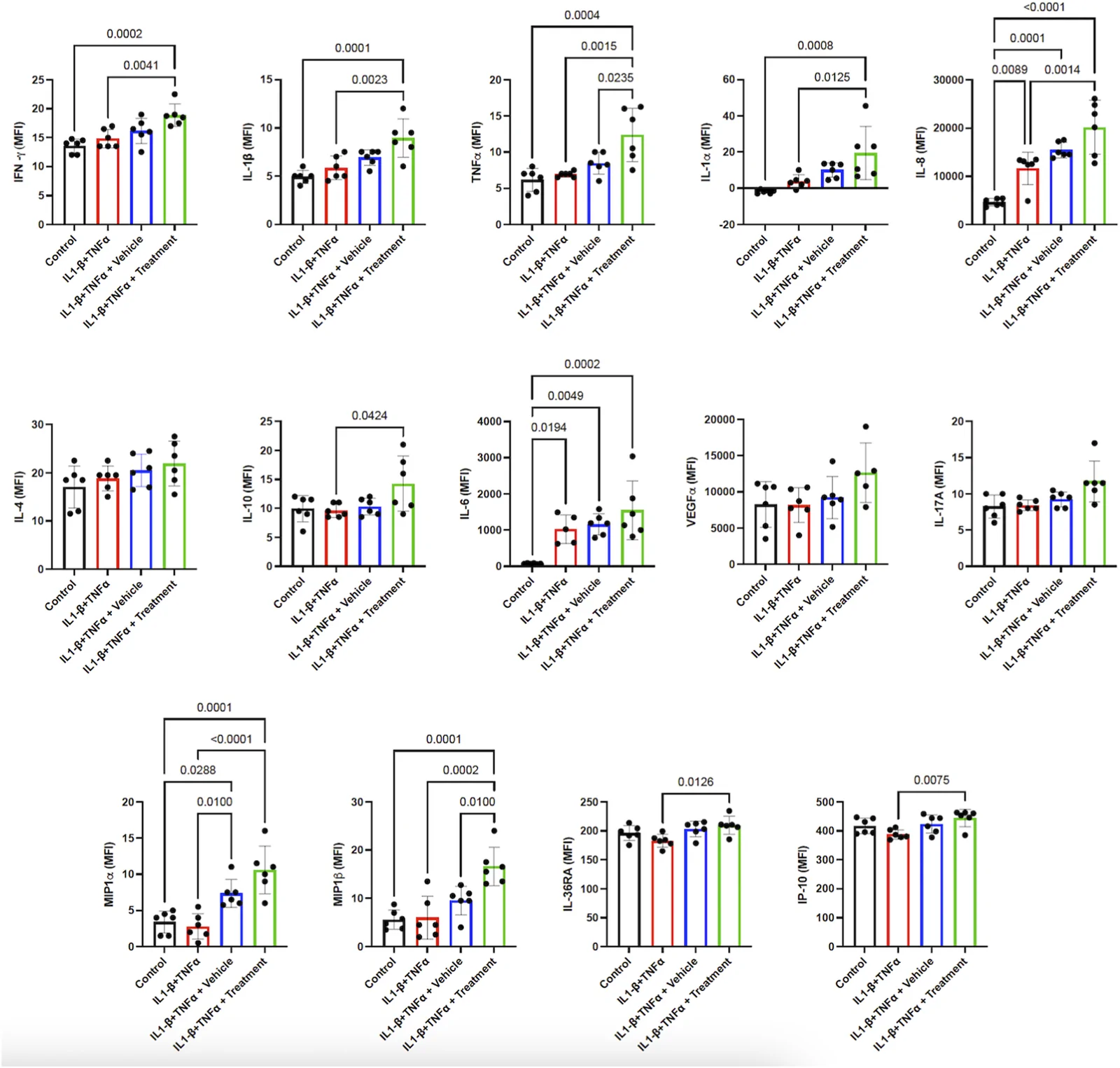
C-1101 promoted pro-inflammatory cytokine secretion in inflamed ovine nucleus pulposus (NP) cells–Similar to AF cells, ovine NP cells were stimulated with IL-1β and TNFα (20 ng/ml) and then treated with vehicle control or C-1101 (50 mg/ml). A non-stimulated control was also used. The stimulated and treated cells were cultured for 24 h, washed with PBS, then cultured for an additional 24 h in complete culture media. Cell-conditioned media was collected at 24 h and frozen at −20° C until it was thawed and analyzed by multiplex bead assay (14-plex).

Annulus fibrosus and nucleus pulposus cells were harvested from intervertebral discs for use in an inflammatory *in vitro* model resembling disc degeneration and related pain. This model analyzes acute changes (24 h of treatment followed by 24 h of conditioning supernatant for analysis) in cellular secretion of cytokines (Figures 2, 3). In AF cells, the group that received the vehicle had small increases in four cytokines (TNFα p = 0.0133, IL-10 p = 0.0396, MIP1α p = 0.0182, IL-36RA p = 0.0043) compared to IL-1β/TNFα stimulated cells, however treatment of AF cells with C-1101 caused a significant increase in 12 out of 14 cytokines (IFNγ p = 0.0011, IL-1β p < 0.0001, TNFα p = 0.0001, IL-1α p = 0.0119, IL-8 p = 0.0179, IL-10 p < 0.0001, IL-6 p = 0.0001, VEGFα p < 0.0001, IL-17A p < 0.0001, MIP1α p = 0.0057, MIP1β p = 0.0013, IL-36RA p = 0.0068). Similar increases are seen in NP cells in the same model. The addition of the vehicle increased the expression of only one cytokine (MIP1α p = 0.01) but when treated with C-1101, 10 cytokines were upregulated (IFNγ p = 0.0041, IL-1β p = 0.0023, TNFα p = 0.0015, IL-1α p = 0.0125, IL-8 p = 0.0014, IL-10 p = 0.0424, MIP1α p < 0.0001, MIP1β p = 0.0002, IL-36RA p = 0.0126, IP-10 p = 0.0075). Further dose titration of C-1101 was performed with AF cells and is shown in Supplementary Document S2. Dorsal root ganglia cells were also treated to C-1101 in this inflammatory *in vitro* model but did not exhibit any significant changes in cytokines released (Supplementary Document S3).

### C-1101 suppressed inflammatory and immune differential gene expression in ovine annulus fibrosus (AF) cells

**FIGURE 4:**
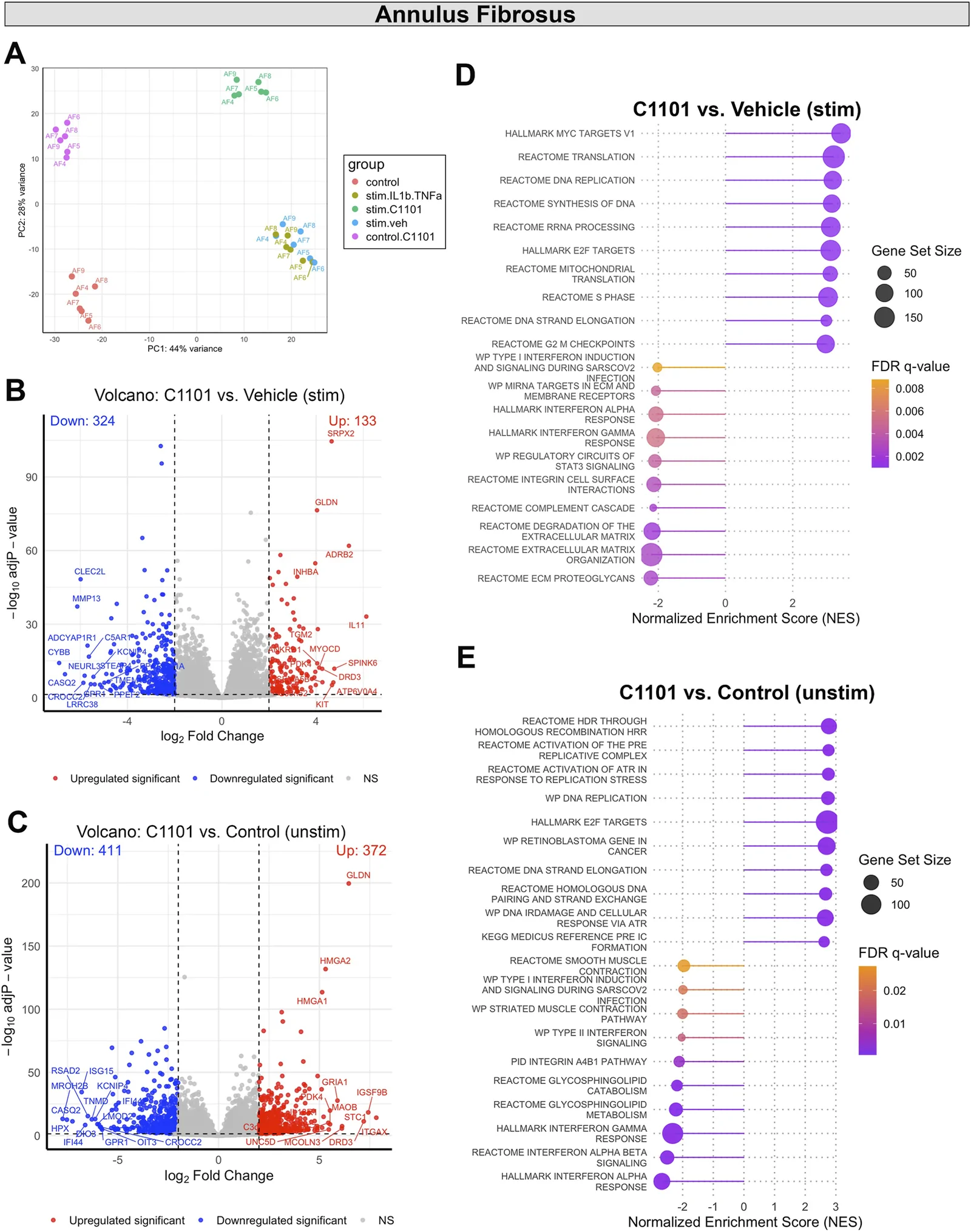
Effects of C-1101 treatment on transcriptome of AF cells. **(A)** PCA (principal component analysis) plot of AF cells from each treatment group, color legend shown on right. Number labels show biological replicate. Top 500 variable genes used for PCA parameters. **(B)** Volcano plot of differential expression results comparing IL1b and TNF-a stimulated C-1101 treated AF cells to vehicle. Transcripts filtered for protein coding genes with annotation. Red dot show significantly upregulated in C-1101, and blue downregulated. Top 15 significant upregulated and downregulated gene names are labeled. Significance defined as Log2 FC>=2 or <=-2 and FDR (false discovery rate) adjusted p-value<=0.05. **(C)** Volcano plot of differential expression results comparing unstimulated C-1101 treated AF cells to unstimulated untreated AF cells. **(D)** GSEA (gene set enrichment analysis) results. Top 20 most upregulated and downregulated pathways comparing C-1101 (stimulated) to vehicle (stimulated). X-axis shows normalized enrichment score, color scale for FDR adjusted p-value and number of genes mapped to pathway shown in circle sizes. Pathways used for analysis include Hallmark, Reactome, PID, KEGG and wikipathways. **(E)** GSEA results comparing unstimulated C-1101 treated AF cells to unstimulated untreated control AF cells. Top 20 pathways shown.

To investigate in depth transcriptomic responses, RNA sequencing was performed on AF cells treated with C-1101. The AF cells were exposed to inflammatory stimulus to mimic the chronic degeneration and immune activation seen in degenerative disc disease. Comparing CBP treated AF cells to the vehicle only treatment (green, blue Figure 4), there were 457 significant DEGs (differentially expressed genes) out of 12,158 protein coding genes with known annotation (Figure 4). The top upregulated and downregulated genes are shown in tables (C, D). Most of the upregulated genes are related to cell cycle and DNA replication. Interestingly, there are many inflammatory pathways that are downregulated after treatment with C-1101, including Type I & Type II interferon signaling, complement, as well as several extracellular matrix (ECM) related pathways.

In addition to treating stimulated AF with C-1101, resting AF were also treated with C-1101 (shown as Trt for treatment) to compare the effects of treatment in a healthy spine to the previously mentioned degenerative environment. The treatment effect on the resting cells was even larger than the stimulated cells, with a total of 783 DEGs. Even in the unstimulated resting AF cells, C-1101 still downregulated several inflammatory pathways such as type I and II interferon responses as well as complement signaling.

### C-1101 suppressed inflammatory and immune differential gene expression in ovine nucleus pulposus (NP) cells

**FIGURE 5:**
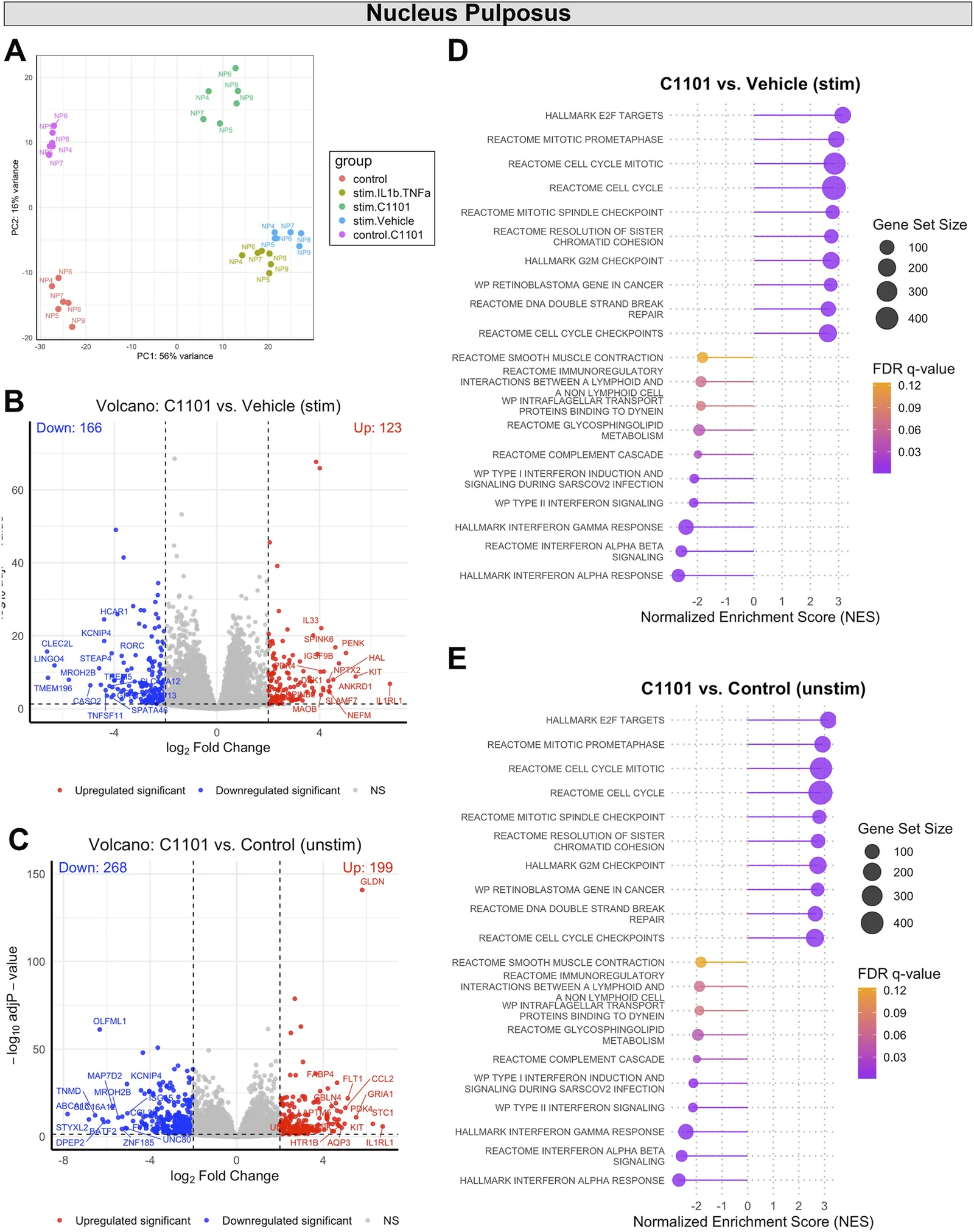
Effects of C-1101 treatment on transcriptome of NP cells. **(A)** PCA plot of n = 3 biological replicates, experimental conditions labeled in color and shown in legend. **(B)** Volcano plot of differential expression results comparing C-1101 (stimulated) NP cells to vehicle (stimulated). Top 15 upregulated (red) and top 15 downregulated (blue) shown. Transcripts filtered for protein coding genes with annotation. **(C)** Volcano plot of differential results comparing unstimulated C-1101 treated NP cells to untreated unstimulated NP cells. **(D)** GSEA results showing top 20 significant pathways in C-1101 treated cells compared to vehicle. **(E)** GSEA results showing top 20 pathways enriched in unstimulated C-1101 treated cells compared to unstimulated untreated NP.

The baseline gene expression pattern was similar between NP and AF cells, with some key differences being the expression of different collagen types, as well as morphological differences. Stimulated NP cells treated with C-1101 have a total of 289 significant DEGs (protein coding, known annotation) (Figure 5 **)**. Pathway analysis of stimulated NP cells treated with C-1101 compared to vehicle was similar to that of the AF C-1101 profile; suppression of type I and II interferon responses and inflammatory pathways, with the addition of several protein kinase pathways that are also suppressed and some additional highly upregulated cell cycle pathways.

In resting NP cells (unstimulated), C-1101 also induced anti-inflammatory changes. There was a total of 467 significant DEGs, again with top downregulated pathways of interferon type I and II, complement and an additional ‘smooth muscle contraction’ category which contains many cytoskeletal proteins.

### C-1101 inhibited immune cell signaling and increased regeneration and metabolic reprogramming in cultured ovine DRG

**FIGURE 6:**
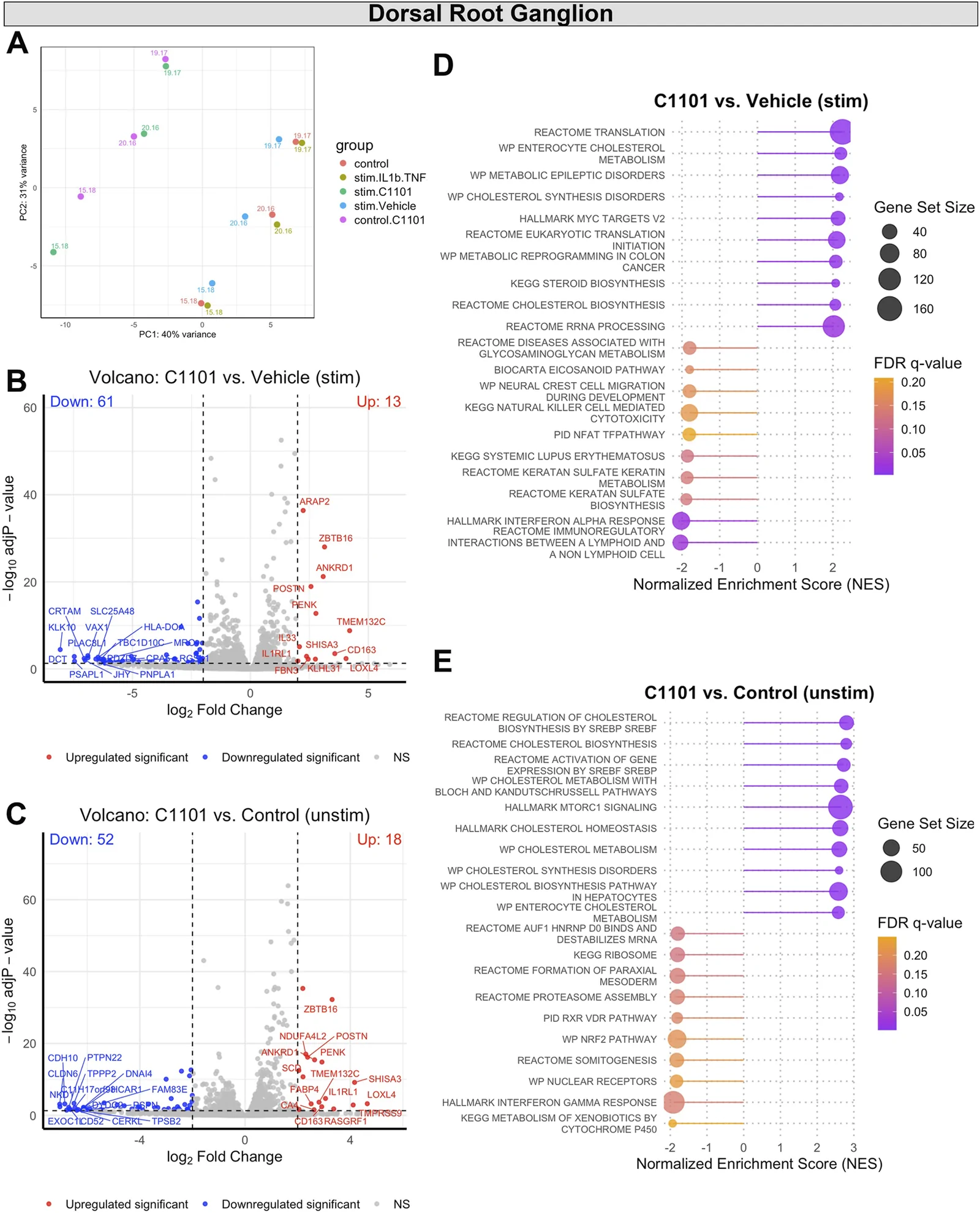
Effects of C-1101 treatment on transcriptome of DRG cells. **(A)** PCA plot of biological replicates of DRG cells, RNA from two biological replicates with the same treatment was combined to achieve sufficient RNA quantity for bulk RNA sequencing. Treatment conditions color labels shown on right. **(B)** Volcano plot of differential expression results comparing C-1101 (stimulated) DRG to vehicle (stimulated). Transcripts filtered for protein coding genes with annotation. **(C)** Volcano plot of differential analysis results comparing unstimulated C-1101 treated DRGs to unstimulated untreated DRG cells. **(D)** GSEA pathway analysis results of top 20 enriched pathways in C-1101 treated stimulated cells compared to vehicle. **(E)** GSEA pathway analysis results of top 20 enriched pathways in unstimulated C-1101 cells compared to untreated unstimulated DRG cells.

Due to the small sample size of the DRGs, two biological replicates were combined for each treatment group resulting in n = 3 replicates analyzed (rather than n = 6 biological replicates collected) in each treatment group. Compared to the AF and NP cells, the IL-1β and TNF-α stimulation has a diminished stimulatory effect on the DRG (Figure 6), potentially due to the lack of immunologically active cells in the dorsal root area.

For the stimulated cells, comparison of C-1101 treatment to the vehicle control treated indicated there were a small number (74 total) of significant DEGs. As with the other two cell types (AF, NP), pathway analysis of DRG indicated downregulation of inflammatory pathways such as interferon gamma, lymphoid cell interaction and NK cell toxicity. In addition, following C-1101 treatment there was a strong upregulation of translation and ribosome activity, and cholesterol and steroid biosynthesis, which coupled with metabolic reprogramming and cell cycle pathways, suggest neuronal growth and increased turnover.

In the unstimulated DRGs, there were also few changes in gene expression (total of 70 significant DEGs). The unstimulated DRGs treated with C-1101 also shared the same downregulation of the interferon gamma, cellular matrix and lymphoid interaction downregulation but with many additional downregulated pathways such as translation and ribosome function and also supression of DNA damage responses. Unstimulated C-1101 treated cells largely shared the same upregulated pathways as the stimulated, with somwhat less of a ‘stress response’ compared to the stimualted C-1101 treated cells.

### C-1101 reduced allodynia and resulted in transient elevation of serum IL-1β and IL-10 and reduction of Iba1 staining in the sciatic nerve in a murine model of peripheral neuropathy

A rodent model of paclitaxel induced allodynia was used to assess the efficacy of C-1101 treatment to reduce neuropathic pain, a component of LSR. Paclitaxel treated mice all developed allodynia, as evidenced by ACT score compared to vehicle control. Treatment with both low (270 mg/kg) and high (540 mg/kg) dose C-1101 significantly reduced paclitaxel-induced allodynia vs. vehicle as assessed by reduction in ACT score at on several days of assessment. The high dose treated group saw a significant pain reduction on day 9 and 11 while low dose treatment caused a significant pain reduction on days 9, 10, 11, and 13.

**FIGURE 7:**
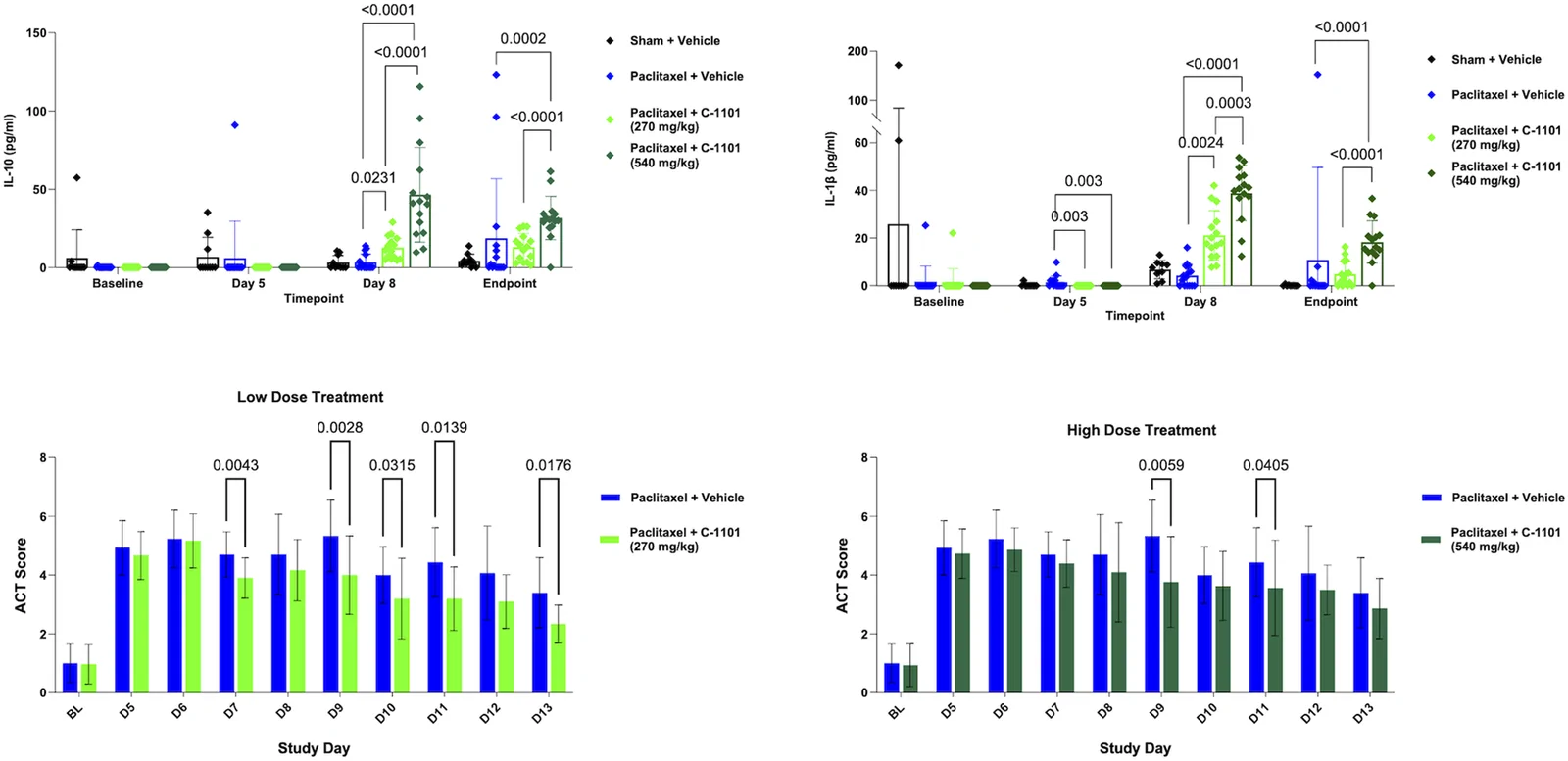
C-1101 reduced allodynia and induced pro-inflammatory cytokine secretion in murine model of peripheral neuropathy. Both low (270 mg/kg) and high (540 mg/kg) dose C-1101 reduced paclitaxel-induced allodynia vs. vehicle by ACT score. Cytokine analyses revealed plasma IL-1β was transiently elevated in C-1101 treatment group on day 8.

To assess the impact of C-1101 treatment on inflammation, circulating cytokine concentrations were also measured in treated mice. These analyses revealed that plasma IL-1β was transiently elevated in both C-1101 treatment groups on day 8 (Figure 7). At endpoint, IL-1β was still elevated in the high dose C-1101 group (p < 0.0001) but had returned to baseline in the low dose C-1101 group. Similarly, IL-10 levels were elevated in both low (p = 0.0231) and high dose (p < 0.0001) groups at day 8, with the low dose group returning to baseline by the endpoint but the high dose group remaining elevated (p = 0.0002).

**FIGURE 8:**
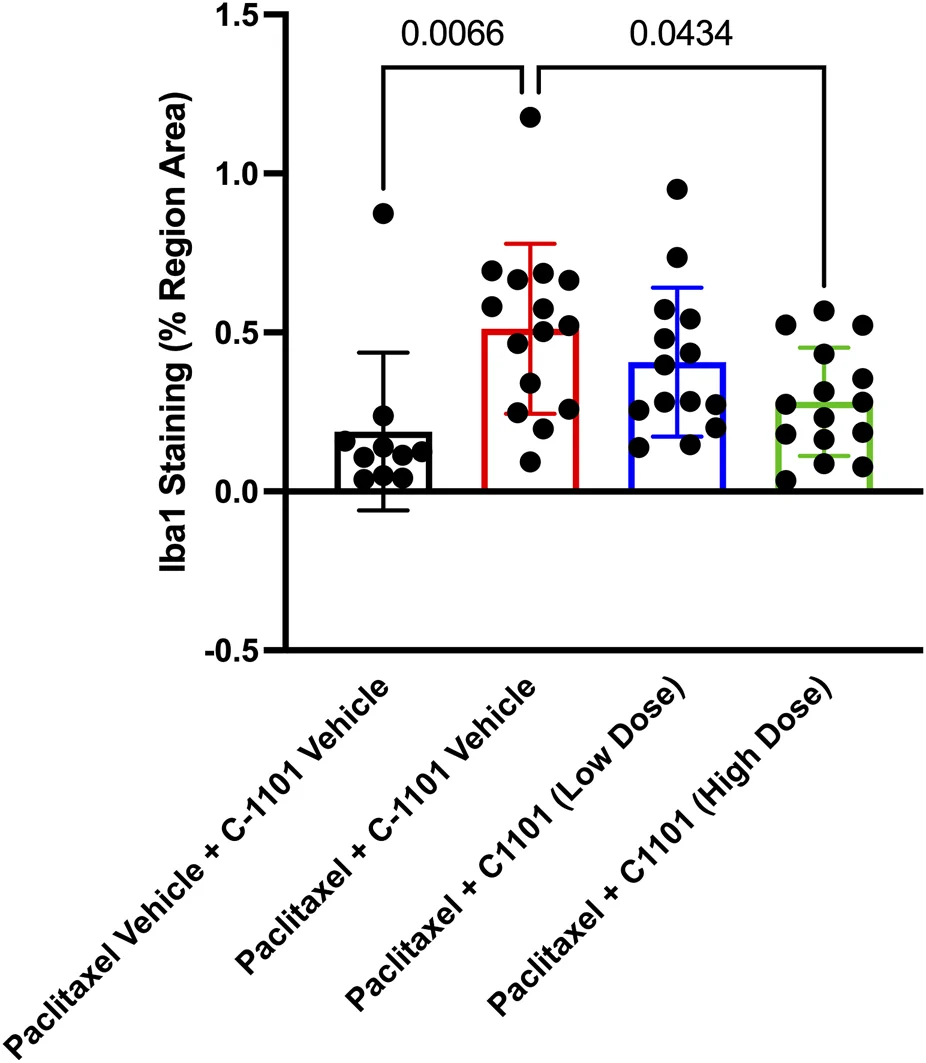
Immunohistochemistry staining indicated high-dose C-1101 reduced Iba-1 in sciatic nerves of paclitaxel-treated mice. Immunostaining was performed with an automated IHC staining system (Ventana Discovery Ultra, Roche) on 5 µm thick paraffin sections paraffin sections. The analysis was performed for Iba-1 IHC for whole section area and results were reported as positive staining percentage of region area (%): [%area = (stained area/total analyzed region area) * 100)]. Data are presented as individual scatter with mean ± SEM, n = 10–15 per group.

Iba1 staining, indicating macrophage and microglial activation, was significantly increased with paclitaxel treatment (paclitaxel+C-1101 vehicle compared to paclitaxel vehicle+C-1101 vehicle p = 0.007) (Figure 8). Administration of high-dose test article C-1101 reduced Iba1 staining in the sciatic nerves of mice compared to paclitaxel+C-1101 vehicle treated mice (p = 0.04).

## Discussion

These studies represent an initial investigation into the biologic and immunologic properties of the multi-protein and platelet-lysate biologic C-1101 in the context of degenerative disc disease as a novel treatment for low back pain. A key finding from these studies was that injections of C-1101 significantly reduced cutaneous pain in a rodent neuropathy model, indicating the potential for C-1101 to also reduce LBP associated with LSR. Mechanistically, our studies demonstrated that C-1101 *in vitro* enhanced production of inflammatory cytokines when exposed to ovine AF and NP cells at the single time point evaluated. The transcriptomic response of AF and NP cells to C-1101 reflected a marked increase in cell replication and translation pathways and a decreased reactome responsiveness to interferons. Cultured DRG treated with C-1101 also displayed increased activity in cell cycle pathways, steroid and cholesterol synthesis with decreased reactome in immune pathways. Taken together, these studies indicate potential therapeutic efficacy, although the mixed inflammatory response induced in cultured cells warrants further investigation of mechanism of action.

Platelet-based therapies have shown prior benefit clinically in musculoskeletal conditions with the potential to alleviate associated pain. A recent meta-analysis evaluating 28 clinical studies on platelet-based therapy indicated effectiveness to reduce pain and improve disability in patients with LBP (Schol et al., 2024). Likewise, several other studies have evaluated the therapeutic effects of PRP in neuropathic pain reporting beneficial results (Kennedy et al., 2025; Wang et al., 2025; Sire et al., 2025; Pretorius et al., 2023). However, the mechanism(s) by which PRP alleviate pain are not fully understood. Platelets are known to be potent modulators (both positive and negative) of inflammation and immune responses (Everts et al., 2020), but new evidence promotes the theory that platelets may elicit their analgesic effects through stimulation of axonal regeneration to ameliorate neuronal hyperexcitability and restoration of neuronal function (Kuffler, 2015). Therefore, the present study builds on prior work expanding the field’s understanding of potential mechanisms of action of platelet-based therapies to reduce pain in IVDD and LSR.

Local inflammation in the AF and NP in IDD is a defining characteristic of disc degeneration and associated pain (Risbud and Shapiro, 2013). It has been well established that AF and NP cells increase production of inflammatory cytokines in degenerated discs (Risbud and Shapiro, 2013). In this study, when AF cells are treated with C-1101 in an inflammatory environment representing IVDD, increased production was seen in both pro-inflammatory (IFNγ, IL-1β, TNFα, IL-1α, IL-6, IL-8, IL-17A) and anti-inflammatory cytokines (IL-10). This pleiotropic milieu can be partially explained by increased cellular metabolism. Upregulation of MIP1α (CCL3) and MIP1β (CCL4) were also seen in AF cells following treatment, which could lead to greater attraction of monocytes and macrophages to local tissues *in vivo*. Similar cytokine changes are seen in NP cells with treatment by C-1101 causing an upregulation in inflammatory cytokines (IFNγ, IL-1β, TNFα, IL-1α, IL-6, IL-8) and anti-inflammatory IL-10. However, slight increases in IL-36RA (anti-inflammatory) and IP-10 (role in IVDD is unknown) are also seen. Likewise, chemokines MIP1α (CCL3) and MIP1β (CCL4) are upregulated in treated NP cells. Overall, mechanistically, C-1101 increased both pro-inflammatory and anti-inflammatory cytokine secretion from AF and NP cells, requiring further evaluation of the proposed analgesic or reparative mechanism(s) of action when considering the reduced allodynia noted in the murine studies.

The transcriptomic responses of AF and NP indicated C-1101 treatment upregulated 133 (AF) and 123 genes (NP) while downregulating 324 (AF) and 166 (NP) genes compared to the vehicle control. The top 10 upregulated pathways in both AF and NP involve almost exclusively cell replication pathways (cell cycle reactomes and checkpoints, DNA synthesis, and translation) while the top 10 downregulated pathways in both AF and NP were associated with decreased interferon responses as well as reduced complement cascade. Independently from NP, the top three downregulated pathways in AF cells emphasize a reduction in the degradation of ECM. These responses in AF and NP cells intimate effects that may ameliorate senescence (Xu et al., 2023; Kelsey, 2024; Li et al., 2025) and apoptotic (Kong and Park, 2025; Xia et al., 2024) causes of IVDD, interferon and complement inflammatory sources of IVDD and associated pain (Xia et al., 2024; Gao et al., 2024), and ECM destruction (Zhang et al., 2022) in the AF region of the IVD. The response of DRGs to C-1101 treatment was more muted. 13 genes were upregulated, and 61 genes were downregulated when compared to vehicle control. In the top 10 upregulated pathways, DRGs also had increased translational pathways. Importantly, the upregulation of steroid and cholesterol synthesis is also observed in these top pathways, indicative of increased neuronal membrane integrity (Wue et al., 2025), reduced hyperexcitability and the associated reduction in hyperalgesia (Amsal et al., 2018; Navia-Pelaez et al., 2021). Taken together, the transcriptomic response in treated AF, NP, and DRG cells suggest a reparative response in IVDD as well as potential to decrease neuropathic pain.

Finally, C-1101 was evaluated *in vivo* in a murine model to assess impact on plasma cytokine analyses, and potential to mitigate pain responses through an acetone cooling test. Treatment with C-1101 resulted in a transient increase in plasma levels of IL-10 and IL-1β at day eight in both low and high dose treatment groups. Cytokine levels in the low dose treated group returned to baseline at endpoint. This transient upregulation could suggest an important temporal increase in IL-1β to initiate inflammation triggering an active response from IL-10 producing immune cells followed by a return to baseline levels to resolve pain hypersensitivity as has been described (Laumet et al., 2020; Interleukin-10 reduces hyperalgesia and the, 2007); however, a direct connection to analgesic activity cannot be concluded from work to date (Soliman and Barreda, 2022). High dose treatment had reduced levels of IL-1β and IL-10 compared to day eight but did not return to baseline levels perhaps indicating a dose-responsive and persistent inflammatory response following treatment, which was reflected in the acetone cooling test as well. Low dose treatment significantly reduced pain response at days nine, 10, 11 and 13 with reductions in high dose only at days nine and 11. The role of IL-1β in nerve regeneration was investigated using a mouse sciatic nerve crush model (Wu et al., 2019). The results of this study suggest that IL-1β promotes physical and functional neural recovery via the nuclear factor-κβ signaling pathway. Others have demonstrated that IL-1β enhances the expression of neurotrophic factors to promote neuronal survival and functional recovery (Zhang et al., 2025). There is a plausible connection between an increase in IL-1β and healing. IL-10 is a well characterized anti-inflammatory cytokine that promotes resolution of inflammation following nerve injury by shifting pro-inflammatory macrophages toward an anti-inflammatory phenotype (Shen et al., 2024). Reduction in pain response by C-1101 and transient increase in inflammatory cytokines with concurrent reduction in microglial activation histologically *in vivo* are in concordance with the *in vitro* AF and NP cytokine findings. However, it is acknowledged that this model does not fully recapitulate chronic lumbosacral radiculopathy or the local inflammatory environment of degenerative disc disease. Additionally, the systemic route of administration performed limits clinical translation of the findings.

Several limitations to study design and opportunities for future directions warrant discussion. The results of this initial *in vitro* study with ovine cells, necessitate further investigation with a larger sample size and a longer duration of evaluation. The present understanding of processes driving local inflammation are currently incomplete; in this context, IL-1β and TNFα were used as potent stimulators of inflammation. However, it is acknowledged that other potential mediators, including inflammatory molecules like nitric oxide and PGE2 as well as the *in vivo* immune response to disc herniations were outside the scope of this study. The cell culture conditions employed here do not completely replicate the spectrum of disease processes observed in IVDD nor do they represent the chronic nature of IVDD progression over time. C-1101 treatment increased both pro-inflammatory and anti-inflammatory cytokines in ovine AF and NP cells and in the murine study. This is suggestive of the proposed reparative or analgesic mechanism(s) of action which reduced allodynia in the murine study but remains exploratory at this stage rather than established. Additionally, the ovine DRG RNA sequencing analysis was limited by necessity to pool samples, resulting in a lower final n = 3 samples per treatment group and adhesion coating of culture dishes was not performed which may have compromised neuron survival. Future work may include whole-cell clamp electrophysiology to provide further mechanistic insight as to whole cell currents and membrane potential following treatment (*e.g.,* testing whether exposure of DRG neurons to C-1101 directly alters neuronal excitability). This could be expanded upon further to compare C-1101 application with conditioned media from C-1101 treated cultures to determine whether C-1101 acts primarily by acting directly on neurons or by altering the surrounding cellular environment.

Demonstration of biological activity in the two animal model systems described here (murine, ovine) lend credence to the translational relevance of this work; however, it is acknowledged that C-1101 is derived from human platelets and plasma, which have been previously investigated in rodent and rabbit models previously (Zunino et al., 2023; Zhang et al., 2024; Yang et al., 2016), although direct comparisons were not performed in this study to support whether level of reactivity is conserved across species as a results of relevant growth factors or conserved receptors. While this study utilized female (sheep) and male (mice) subjects, tissues derived from only one sex were evaluated in each study, demonstrating biological activity in both sexes but limiting the direct comparison and delineation of sex as a biological variable or sex-related effects. The rodent paclitaxel-induced peripheral neuropathy model is acknowledged to not fully recapitulate chronic LSR or the local inflammatory environment of DDD, limiting direct translation of findings to clinical patients at this time. In addition, as neuropathies may present with varying pain phenotypes, further evaluation of mechanical and thermal hypersensitivity in the rodent model is indicated to determine whether specific neuronal subtypes are more susceptible to C-1101 treatment. Furthermore, while murine models are useful, large animal models are generally considered to provide the strongest translational data. The ovine preclinical model was selected for its tissue availability as well as similarities in disc tissue volume, thickness and loading forces comparable to humans. Further studies in large animal models are needed to elucidate the timing, amount of treatment needed and to determine the long term clinical therapeutic effect for C-1101. However, the findings in this study indicate a beneficial therapeutic effect on pain management in IVDD and LSR and encourages further studies in large animal and *in vitro* models to evaluate mechanism of action further. Clinical studies are ongoing to assess the safety of epidural administration of C-1101 (ClinicalTrials.gov ID NCT07264270), and additional work to evaluate the potential efficacy in treatment of lumbosacral radiculopathy represents a future direction (Sorkin and Yaksh, 2018).

## Conclusion

In summary, the protein and platelet derived product, C-1101, reduced allodynia in a murine peripheral neuropathy model and had potent immunomodulatory properties on cell lines (*e.g.,* AF, NP, DRG) relevant to low back pain associated with intervertebral disc degeneration. Gene expression analyses revealed acute upregulation of cell replication and translation pathways from AF and NP cells, and downregulation of key pathways related to inflammation (namely, interferons), ECM and cell signaling. These findings support potential translation of C-1101 to mitigate low back pain and associated inflammation, warranting further *in vivo* evaluation in the context of chronic lumbar radiculopathy.

## Data Availability

The datasets presented in this study can be found in online repositories. The names of the repository/repositories and accession number(s) can be found in the article/Supplementary Material.
